# xViTCOS: Explainable Vision Transformer Based COVID-19 Screening Using Radiography

**DOI:** 10.1109/JTEHM.2021.3134096

**Published:** 2021-12-08

**Authors:** Arnab Kumar Mondal, Arnab Bhattacharjee, Parag Singla, A. P. Prathosh

**Affiliations:** Amar Nath and Shashi Khosla School of Information TechnologyIndian Institute of Technology Delhi 28817 New Delhi 110016 India; UQ-IITD Academy of ResearchIndian Institute of Technology Delhi 28817 New Delhi 110016 India; Department of Computer Science and EngineeringIndian Institute of Technology Delhi 28817 New Delhi 110016 India; Department of Electrical Communication EngineeringIndian Institute of Science (IISc) Bangalore 560 India

**Keywords:** AI for COVID-19 detection, CT scan and CXR, deep learning, vision transformer

## Abstract

*Objective:* Since its outbreak, the rapid spread of COrona VIrus Disease 2019 (COVID-19) across the globe has pushed the health care system in many countries to the verge of collapse. Therefore, it is imperative to correctly identify COVID-19 positive patients and isolate them as soon as possible to contain the spread of the disease and reduce the ongoing burden on the healthcare system. The primary COVID-19 screening test, RT-PCR although accurate and reliable, has a long turn-around time. In the recent past, several researchers have demonstrated the use of Deep Learning (DL) methods on chest radiography (such as X-ray and CT) for COVID-19 detection. However, existing CNN based DL methods fail to capture the global context due to their inherent image-specific inductive bias. *Methods:* Motivated by this, in this work, we propose the use of vision transformers (instead of convolutional networks) for COVID-19 screening using the X-ray and CT images. We employ a multi-stage transfer learning technique to address the issue of data scarcity. Furthermore, we show that the features learned by our transformer networks are explainable. *Results:* We demonstrate that our method not only quantitatively outperforms the recent benchmarks but also focuses on meaningful regions in the images for detection (as confirmed by Radiologists), aiding not only in accurate diagnosis of COVID-19 but also in localization of the infected area. The code for our implementation can be found here - https://github.com/arnabkmondal/xViTCOS. *Conclusion:* The proposed method will help in timely identification of COVID-19 and efficient utilization of limited resources.

## I. Introduction

### A. Background

The novel COronaVIrus Disease 2019 (COVID-19) is a viral respiratory disease caused by Severe Acute Respiratory Syndrome COronaVirus 2 (SARS-CoV2). The World Health Organization (WHO) has declared COVID-19 a pandemic on 11 March 2020 [1]. This has pushed the health systems of several nations to the verge of collapse. It is, therefore, of utmost importance to screen the positive COVID-19 patients accurately for efficient utilization of limited resources. Two types of viral tests are currently popularly used to detect COVID-19 infection: Nucleic Acid Amplification Tests (NAATs) [2] and Antigen Tests [3]. NAATs can reliably detect SARS-CoV-2 and are unlikely to return a false-negative result of SARS-CoV-2. NAATs can use many different methods, among which Reverse Transcription Polymerase Chain Reaction (RT-PCR) is the most preferred test for COVID-19 due to its high specificity and sensitivity [4]. However, this test is expensive as it has an elaborate kit and time-consuming. An RT-PCR test uses nose or throat swabs to detect SARS-CoV-2 and requires trained professionals instructed for the RT-PCR kit to carry out the RT-PCR test. RT-PCR requires a complete set-up that includes the trained practitioners, laboratory, and RT-PCR machine for detection and analysis.

### B. Scope and Contributions

Motivated by the success of the Deep Learning in diagnosing respiratory disorders [5], several recent works have proposed the use of chest radiography images (X-ray and Computed Tomography, CT) as alternate modality to detect COVID-19 positive cases [6]–[12] (Elaborated in Sec. II). Unlike in the chest CT/X-ray of a healthy person, the lungs of COVID-19 affected patients show some visual marks like ground-glass opacity and/or mixed ground-glass opacity, and mixed consolidation [6].

While there has been a large body of literature on use of Deep Learning for Covid detection, most of them are based on Convolutional Neural Networks (CNNs) [12]–[15]. CNN, albeit powerful, lacks a global understanding of images because of its image-specific inductive biases. To capture long-range dependencies, CNNs require a large receptive field, which necessitates designing large kernels or immensely deep networks, leading to a complex model challenging to train. Recently, Vision transformers [16] have provided an alternative framework for learning tasks and overcome the issues associated with convolutional inductive bias as they can learn the most suitable inductive bias depending on the task at hand. Motivated by this, in this work, we propose to employ a vision transformer (ViT) based transfer learning method to detect COVID-19 infection from the chest radiography (X-ray and CT scan imaging). Specifically, the below are our contributions:

1) We propose a vision transformer based deep neural classifier, xViTCOS for screening of COVID-19 from chest radiography.
2) We provide explanability-driven, clinically interpretable visualizations where the patches responsible for the model’s prediction are highlighted on the input image.
3) We employ a multi-stage transfer learning approach to address the problem of need for large-scale data.
4) We demonstrate the efficacy of the proposed framework in distinguishing COVID-19 positive cases from non-COVID-19 Pneumonia and Normal control using both chest CT scan and X-ray modality, through several experiments on benchmark datasets.

## II. Related Work

### A. COVID-19 Detection Using Chest CT

Chest Computed Tomography (CT) imaging has been proposed as an alternative screening tool for COVID-19 infection [6], [7]. In [17] multiple features, such as Volume, Radiomics features, Infected lesion number, Histogram distribution and Surface area are extracted first from the CT images following which a deep forest algorithm, consisting of cascaded layers of multiple random forests, is used for discriminative feature selection and classification.

The work in [13] performs a comparative study by exploiting transfer-learning to optimize 10 pre-trained CNN models viz AlexNet [18], VGG-16 [19], VGG-19 [19], SqueezeNet [20], GoogleNet [21], MobileNet-V2 [22], ResNet-18 [23], ResNet-50 [23], ResNet-101 [23], and Xception [24] on CT-scan images to differentiate between COVID-19 and non-COVID-19 cases. As per the results reported in [13], ResNet-101 and Xception achieve best performance. [25] segment out candidate infection regions from the pulmonary CT image set using a 3D CNN segmentation model and categorize these segments into the COVID-19, IAVP, and irrelevant to infection (ITI) groups, together with the corresponding confidence scores, using a location-attention classification model. COVNet [26] is a ResNet50 based CNN architecture that takes as input a series of CT slices and compute features from each slice of the CT series, which are combined by a max-pooling operation, and the resulting feature map is fed to a fully connected layer to generate a probability score for each class. Ref. [27] uses a pre-trained EfficientNet as the backbone and extracts features from each slice of CT data, and makes a binary prediction. Next, the slice level predictions are combined using a multi-layer perceptron (MLP) to make a final prediction at the patient level. COVIDNet-CT [15] on the other hand offers architectural diversity, selective long-range connectivity, and lightweight design patterns. Ref. [28] proposes Contrastive COVIDNet which is built upon the COVIDNet [11] architecture by introducing domain specific batch normalization layers along with a cross entropy classification and a contrastive loss. In [29] a custom CNN model is built with two separate lines of forward pass and deep feature aggregation to classify COVID and non-COVID. The network is trained to work both on CT and X-ray data. It employs a deep feature aggregation strategy by aggregating layer outputs from varying depths following a classifier network. ResGNet-C [30] exploits Graph Convolution Network (GCN) [31] to perform binary classification task using the Resnet-101 [23] extracted features. Ref. [32] proposes an hybrid model based on deep features and Parameter Free BAT (PF-BAT) optimized Fuzzy K-nearest neighbor (PF-FKNN) classifier for COVID-19 prognosis.

### B. COVID-19 Detection Using Chest X-Ray

Although chest-CT has more sensitivity as compared to RT-PCR [8], [9], associated cost and resource constraints makes routine CT screening for COVID-19 detection a less accessible solution to the third world’s teeming millions. Therefore, digital X-ray based Covid detection is considered an easily accessible alternative.

In [34] the authors propose a two-stage pipeline for binary classification. In the first stage, the significant lung region is cropped from the chest X-ray images using a bounding box segmentation. In the second stage, a GAN inspired class – inherent transformation network is used to generate two class inherent transformations which are then used to solve a four-class classification problem using a CNN. However, as the number of classes increase, the number of generators to be trained in the second stage of this method will increase accordingly, making it difficult to scale for multi class classification. COVID-Net [11] leveraged a human-machine collaborative design strategy to produce a network architecture tailored for COVID-19 detection from chest X-ray images. CoroNet [12] uses Xception [24] backbone for extracting CXR image features which are classified using a multi-layer perceptron (MLP) classification head. CovidAID [35] finetunes a pretrained CheXNet [5]. Ref. [36] proposes a novel architecture with multiscale attention-based generation augmentation and guidance for training a CNN model for COVID-19 diagnosis. The multi-scale attention features are computed from the intermediate feature maps of a Resnet-50 [23] based feature extractor and are combined with the final feature map to obtain the predictions. Ref. [37] proposes another attention based CNN model incorporating a teacher-student transfer learning framework for COVID-19 diagnosis from Chest X-ray and CT images. CHP-Net [38] consists of three networks: a bounding box regression network to extract bi-pulmonary region coordinates, a discriminator deep learning model to predict a differentiating probability distribution, and a localization deep network that represents all potential pulmonary locations. In [10] the authors propose using shape dependent Fibonacci p patterns to extract features from chest X-ray images and then apply conventional machine learning algorithms. Ref. [39] first extracts orthogonal moment features using Fractional Multichannel Exponent Moments (FrMEMs). Next, the most significant features are selected using a differential evolution based modified Manta-Ray Foraging Optimization (MRFO). Finally a KNN classifier is trained to distinguish COVID-19 positive cases from negative cases.

### C. Transformers and Self Attention in Vision

Images can be naively represented using a sequence of pixels for analysis using transformers but that would lead to huge computational expenses with a quadratic increase in costs. This has led to a number of approximations. For example, [40] used self attention in local neighbourhoods of query pixels instead of performing calculation globally with the entire rest of the image. Such local multi head attentions can be shown to replace convolutions ([41], [42], [43]). Ref. [44] proposed Sparse Transformers where scalable approximations to global self attention are employed for images. Ref. [45] used an alternative way of scaling attention by applying them in blocks of varying sizes. Ref. [46] applies full attention after extracting patches of size $2 \times 2$ from the input image. The use of small patch size, however, enables the model to be used only for small resolution images. Other than transformers, a number of researchers have combined convolutional neural networks with different forms of self attention. Ref. [47] uses attention to augment feature maps for image classification. A lot of work has come up where the authors have used self attention for further processing the output of a CNN for a number of tasks including, object detection ([48]) image classification ([49]), video analysis ([50], [51]), etc. A recent approach by [52] applies Transformers to pixel level patches after reducing image resolution and color space. The model named image GPT is trained like a generative model whose representations are then fine tuned or linearly probed for performing classification tasks.

## III. Proposed Method

**FIGURE 1.**
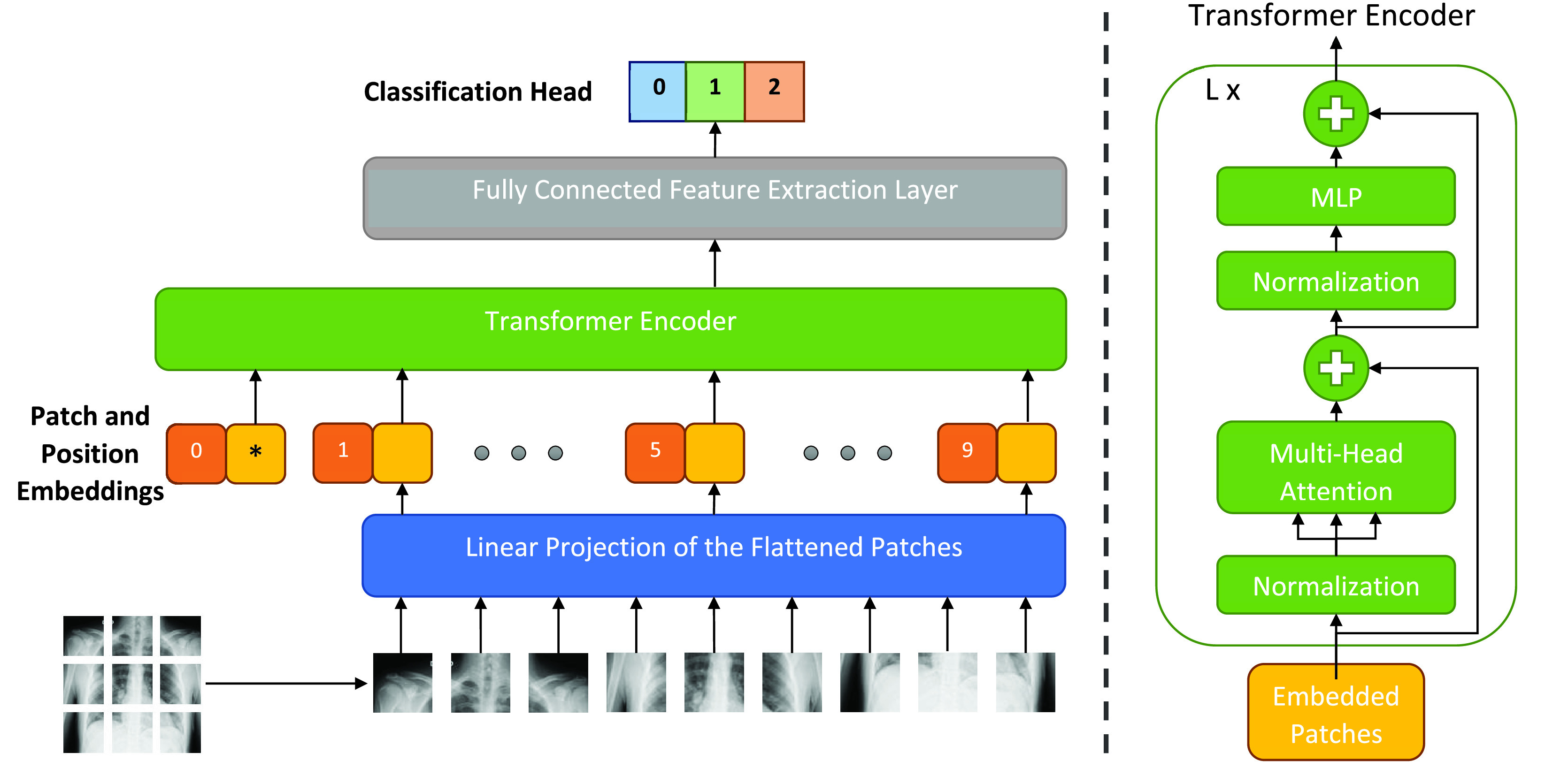
xViTCOS: Illustration of our proposed network for COVID-19 detection using chest radiography (CT scan/CXR image). The input image is split into equal-sized patches and embedded using linear projection. Position embedding are added and the resulting sequence is fed to a Transformer encoder [33].

Unlike the existing methods that incorporate CNNs, we propose a vision transformer (ViT) [16] based model for automated COVID-19 screening and call it xViTCOS, illustrated in Figure 1. Since we use xViTCOS on two chest radiography modalities CT scan images and chest X-ray images, we refer to them as xViTCOS-CT and xViTCOS-CXR respectively.

### A. Vision Transformers

A Vision Transformer [16] is a deep neural model that adapts the attention-based transformer architecture [33] prevalent in the domain of natural language processing (NLP) to make it suitable for pattern recognition in visual image data. While the original transformer architecture comprises of an encoder and a decoder, vision transformer is an encoder-only architecture. For non-sequential image analysis tasks, like image classification, the input image, $\boldsymbol {x} \in \mathbb {R}^{H\times W\times C}$ is broken down into $N$ image patches, $\boldsymbol {x}_{p}^{(i)} \in \mathbb {R}^{P\times P\times C},\,\,\text {where } i \in \{1, \cdots N\}$, and each patch is of shape $P\times P$ in 2-D, $C$ denotes the number of channels (e.g. $C=3$ for RGB images) and $N = \frac {H\times W}{P\times P}$. These patches derived from the image is then effectively used as a sequence of input images for the Transformer. The input patches are first flattened and then mapped to a D dimensional latent vector through a trainable linear projection layer, leading to the generation of patch embeddings. Throughout its layers, the transformer maintains a constant latent vector size of D. Similar to the [class] token in BERT [53], a learnable embedding is embedded to the sequence of the patch embeddings ( $\boldsymbol {Z}^{0}_{0} = \boldsymbol {x}_{class}$). The final transformer layer state corresponding to this class token, $\boldsymbol {z}^{0}_{L}$, represents in a compact form the classification information that the model is able to extract from the image( $\boldsymbol {y}$). The classification head is attached to $\boldsymbol {z}^{0}_{L}$ during both pre-training and fine-tuning. In order to retain crucial positional information, standard learnable 1D position embeddings are added to the patch embeddings. The final resulting sequence is provided as input to the encoder. During pre-training, an MLP is used to represent the classification head and it is replaced by a single linear layer during the fine-tuning stage. As illustrated in the Figure 1, the transformer encoder of a vision transformer consists of alternating layers of multiheaded self-attention (MSA) and MLP blocks. Layernorm (LN) is applied before every block, and residual or skip connections after every block. The workings of the vision transformer can be mathematically described in Equations below:

\begin{align*} \boldsymbol {z}_{0}=&\left [{\boldsymbol {x}_{class}; \boldsymbol {x}_{p}^{1}\boldsymbol {E};\boldsymbol {x}_{p}^{2}\boldsymbol {E};\cdots \boldsymbol {x}_{p}^{N}\boldsymbol {E}}\right] + \boldsymbol {E}_{pos} \tag{1}\\ \boldsymbol {z}'_{l}=&\text {MSA}\left ({\text {LN}\left ({\boldsymbol {z}_{l-1}}\right)}\right) + \boldsymbol {z}_{l-1}, \quad \forall l = 1 \cdots L \tag{2}\\ \boldsymbol {z}_{l}=&\text {MLP}\left ({\text {LN}\left ({\boldsymbol {z}_{l}'}\right)}\right) + \boldsymbol {z}_{l}',\quad \forall l = 1 \cdots L \tag{3}\\ \boldsymbol {y}=&\text {LN}\left ({\boldsymbol {z}_{L}^{0}}\right) \tag{4}\end{align*}

where $\boldsymbol {E} \in \mathbb {R}^{(P^{2}C)\times D} \,\,\text {and} \,\,\boldsymbol {E}_{pos} \in \mathbb {R}^{(N+1)\times D}$

### B. Inductive Bias in ViT

Unlike CNN based models that impose inherent bias such as translation invariance and a local receptive field, vision transformer (ViT) [16] has much less image specific inductive bias. This is because ViT treats an image as a sequence, hence loses any structural and neighborhood information a CNN can easily recognize. Although MLP layers are local and translationally equivariant, the self-attention layers are global. The only mechanism that adds inductive bias and provides structural information about the image to the encoder are the position embeddings, that are concatenated with the patch embeddings. Without those, the Vision Encoder might find it difficult to make sense of the image patch sequence. Consequently, ViT does not generalize well when trained using insufficient amount of data. This might be a bit discouraging but the entire status quo changes as the size of the dataset increases. The large size of the training dataset overshadows the dependence of the model on inductive bias for generalization. As can be expected, using a ViT model pretrained on a large training dataset under a transfer learning framework on a smaller target dataset leads to improved performance. Next, we propose a multi-stage transfer learning strategy.

### C. Multi-Stage Transfer Learning

A domain and a task are the two main components of a typical learning problem. For the specific case of a supervised classification problem, the domain, $\mathcal {D}$ might be defined as the tuple of the feature space, $\mathcal {X}$, and the marginal feature distribution, $P(X)$, i.e. $\mathcal {D} = \langle \mathcal {X}, P(X)\rangle $. The task, $\mathcal {T}$ is a tuple of label space, $\mathcal {Y}$, and the posterior of the labels conditioned on features, $P(Y|X)$, i.e. $\mathcal {T} = \langle \mathcal {Y},P(Y|X)\rangle $. Any change in either of the two components of a machine learning problem would cause severe degradation in the performance of the trained model and necessitates rebuilding the model from scratch. Transfer Learning is a way to combat this issue.

Given a source domain, $\mathcal {D}_{s}$ and a corresponding task, $\mathcal {T}_{s}$, and a target domain, $\mathcal {D}_{t}$ and a corresponding task, $\mathcal {T}_{t}$, the objective of transfer learning is to improve the performance of a machine learning model in $\mathcal {D}_{t}$ using the knowledge acquired in $\mathcal {D}_{s}$ and $\mathcal {T}_{s}$ [54]. Transfer learning has played a significant role in the facilitating the use of deep learning in numerous applications [55]–[57]. In this work, we empirically demonstrate how knowledge transfer is equally effective for vision transformer based framework in medical image classification.

In the current problem, the target domain consists of chest radiography image data i.e., for xViTCOS-CXR, the target data is the COVID-19 CXR dataset and for the xViTCOS-CT model, the target data consists of the COVIDx-CT-2A dataset [58] with three classes – COVID-19 Pneumonia, non-COVID-19 Pneumonia, and normal.

The first source domain $\mathcal {D}_{S_{1}}$ that our proposed ViT model is trained on consists of a large-scale general-purpose image dataset, ImageNet [59]. Since effective ViT training demands access to a sufficiently large number of data points, we choose a model which is pretrained on ImageNet-21k [59] $\left ({\mathcal {T}_{S_{1}}}\right)$ in a self-supervised manner and later finetuned on ImageNet-2012 [60] $\left ({\mathcal {T}_{S_{2}}}\right)$. This pre-training aims to ensure that the model learns to extract crucial but generic image representations to classify natural images.

The underlying distribution of clinical radiographic images is vastly different from an unconnected set of natural images like those in ImageNet, and distributional divergence is very high between the two domains. Hence in cases where the target dataset is of insufficient capacity, the pre-trained ViT model might find it highly difficult to bridge the domain shift between the learned source domain and the unseen target domain. However, with a sufficient number of training examples available from the target domain, the ViT model can overcome the gap between these two domains. Keeping this in mind, an intermediate stage of knowledge transfer is used in this paper to train our proposed model depending on the size of the target domain training data. The primary goal of this stage of transfer learning is to help the ViT model, pre-trained on a generic image domains $\mathcal {D}_{S_{1}}, \mathcal {D}_{S_{2}}$, to learn chest radiography specific representations to overcome the existing domain shift. In order to achieve this, we further finetune the pre-trained ViT model on a large collection of chest radiographic data $\left ({\mathcal {D}_{S_{3}}}\right)$ [61] after replacing its existing classification head with one suitable for the corresponding classification task $\left ({\mathcal {T}_{S_{3}}}\right)$.

With the COVIDx-CT-2A dataset [58] a moderate-sized dataset (refer to Table 1), xViTCOS-CT model was able to overcome the domain shift and achieved state-of-the-art performance without the need for the intermediate finetuning stage. However, due to a limited number of COVID-19 CXR images (refer to Table 2), an intermediate stage of knowledge transfer was employed to improve the performance of xViTCOS-CXR model. A publicly available large-scale CXR dataset, CheXpert [61] was used, and xViTCOS-CXR was finetuned to classify five medical conditions (Atelectasis, Cardiomegaly, Consolidation, Edema, and Pleural Effusion) and the case of no finding on that dataset. Following this, the existing classification head of the ViT network was replaced by a new head suited for the particular target task, i.e., COVID-19 detection, and the model was further finetuned on the target domain. Refer to supplementary material for an ablation study to understand the impact of multi-stage transfer.

**TABLE 1:** Summary of COVIDx CT-2A Dataset [58]

Split	Normal	Pneumonia	COVID-19	Total
Train	35996	25496	82286	143778
Validation	11842	7400	6244	25486
Test	12245	7395	6018	25658

**TABLE 2:** Summarized Description of CXR Dataset

Split	Normal	Pneumonia	COVID-19	Total
Train	1079	3106	1726	5911
Validation	270	777	432	1479
Test	234	390	200	824

### D. Implementation Details

A number of Vision Transformers architectures have been proposed in literature. In this paper we have tested our algorithm on architectures proposed in [53] and [16] over the task of classification on the Chest X-Ray dataset. A detailed study on all the architectures tested, namely ViT-B/16, ViT-B/32, ViT-L/16 and ViT-L/32, and the results obtained has been added in the supplementary. On the basis of classification performance and computational expense, we choose the ViT-B/16 network as the most suitable amongst those tested for further experimentation. For further details, please refer to the Supplementary. ViT-B/16 architecture has the following configuration- Patch size: $16\times 16$, Fraction of the units to drop for dense layers (Dropout rate): 0.1, Dimensions of the MLP output in the transformers: 3072, Number of transformer heads: 12, Number of transformer layers: 12, Hidden size: 768. The model parameters are initialized with the parameters of a model pretrained on ImageNet-21k [59] and fine-tuned on ImageNet-2012 [60].

While training xViTCOS-CXR, for the intermediate finetuning step using CheXpert [61], we use standard binary cross-entropy loss. This is because the classification task using CheXpert is a multi-label classification problem. Finally, while finetuning in the target COVID-19 CXR images, categorical cross-entropy loss is used to solve a multi-class classification problem. While training xViTCOS-CT, we utilize categorical cross-entropy. We use Keras [62] with Tensorflow [63] backend and vit-Keras.^1^^1^https://github.com/faustomorales/vit-keras

## IV. Experiments and Results

### A. Datasets

Some of the existing works validate their methods using private datasets [30], and several other works [12], [14], [15], [35] combine data from different publicly available sources. While combining data from different public repository, researchers should be careful to avoid duplication as a contributor might upload the same image to many of the repositories. Another interesting way to mitigate the issue of data scarcity is through generative data augmentation where a neural generative framework [64]–[67] is trained to generate novel data samples. However in this work, we use the datasets described in the next section. We have rerun the codes of the baseline models using same dataset and same split to ensure a fair comparison.

#### 1) CT Scan Dataset

To demonstrate the efficacy of xViTCOS-CT, we use COVIDx CT-2A dataset [58], derived from several public repositories [68]–[75]. This dataset contains 194,922 CT scans from 3,745 patients across the globe with clinically verified findings. Table 1 summarizes the important statistics of COVIDx CT-2A dataset.

#### 2) Chest X-Ray Dataset

To benchmark xViTCOS-CXR against other deep learning based methods for COVID-19 detection using CXR images, we construct a custom dataset consisting of three cases: Normal, Pneumonia, and COVID-19. Like in [12], [35], Normal and Pneumonia CXR images were obtained from the Kaggle repository ‘Chest X-Ray Images (Pneumonia)’ [76], which is derived from [77]. COVID-19 images were collected from the Kaggle repository ‘COVIDx CXR-2’ [78], which is a compilation of several public repositories [79]–[84].

COVIDx-CXR-2 [78] provides only Train-Test split of the data. To automatically select the best model based on validation-set performance, we split Training set in 80:20 ratio as train and validation set. This would have caused huge class imbalance in the validation set as ‘Chest X-Ray Images (Pneumonia)’ [77] contains only 8 images per class in the validation set. Therefore, we combine the training and validation split and reconstruct the training and validation split in 80:20 ratio. Table 2 summarizes split-wise image distribution. Note that, we have kept the test split intact in both the datasets to prevent patient-wise information leakage as multiple images for the same patient could be present in the dataset.

### B. Data Preprocessing and Augmentation

#### 1) CT Images

COVIDx CT-2A dataset [58] provides bounding box annotations for the body regions within the CT images. To standardize the field-of-view in the CT images, we crop the images to the body region using this additional information. Next each cropped image is resized to a fixed size of $224 \times 224$ pixels. To improve generalizability of the model, we augment the training data on the fly by applying random affine transformations such as rotation, scaling and translation, random horizontal flip and random shear.

#### 2) CXR Images

In the compiled dataset, the chest X-ray images are of various sizes. To fix this issue, all the images were resized to a fixed size of $224 \times 224$ pixels. Again as in the case of CT images, to improve the generalizability of the model, we apply the same sets of augmentation techniques (refer to Section IV-B.1). In addition, we apply random zoom in and zoom out, and random channel shift.

### C. Quantitative Results

To quantify and benchmark the performance of xViTCOS, we compute and report Accuracy, Precision (Positive Prediction Value), Recall (Sensitivity), F1 score, Specificity, and Negative Prediction Value (NPV) as defined and compared in the standard literature such as [14], [32].

#### 1) xViTCOS-CT

Table 3 presents the overall accuracy of xViTCOS-CT on the test split of COVID-CT-2A dataset [58]. As can be observed, the proposed method achieves the best accuracy score of 98.1%, surpassing the current state of art methods. Next, we discuss the precision, recall, specificity, PPV, NPV, and F1-scores attained by the model on test COVID CT images and interpret their significance in determining the classification caliber of the model. From table 3, it can be observed that xViTCOS-CT achieves a high value of recall or sensitivity at 96%, implying that a small proportion of pneumonia cases caused due to COVID-19 are incorrectly classified as having non-COVID-19 origin. This implies a significantly low number of false-negative cases, which is a highly sought-after characteristic in a medical data classifier as in such cases, a false negative situation may lead to denial or delay of treatment to a person genuinely infected by the disease. The proposed method also attains a high precision or positive predictive value of 96% for COVID-19 cases, implying a little chance of the model classifying a non-COVID case as having a COVID-19 origin. However, the usefulness of our proposed method lies in the fact that it achieves the highest F1 scores for all the classes, implying that in terms of both precision and recall, the proposed method is the most balanced amongst all the baseline models. Also, it is well able to differentiate between the normal and Pneumonia cases of patients as well. Similarly, we can see that the proposed model attains high specificity and NPV values of 98.8% for the COVID-19 case, implying that false positives are also very low. This is a useful characteristic in clinical scenarios since the model correctly rejects all the negative cases, facilitating efficient utilization of limited resources.

**TABLE 3:** Comparison of Performance of xViTCOS-CT on CT Scan Dataset Against State-of-the-Art Methods

Method	Class Label	Precision	Recall	F1-score	Specificity	NPV	Overall Accuracy
Resnet + Location Attention [25]	Normal	0.920	0.989	0.954	0.922	0.989	0.932
	Pneumonia	0.963	0.799	0.873	0.987	0.924	
	COVID-19	0.906	0.955	0.930	0.969	0.986	
	Weighted Avg.	0.929	0.926	0.925	0.952	0.970	
	Macro Avg.	0.930	0.914	0.919	0.959	0.966	
COVIDNet-CT [15]	Normal	0.958	0.987	0.973	0.957	0.986	0.949
	Pneumonia	0.981	0.805	0.884	0.989	0.942	
	COVID-19	0.906	0.988	0.945	0.960	0.995	
	Weighted Avg.	0.952	0.935	0.941	0.967	0.975	
	Macro Avg.	0.948	0.927	0.934	0.969	0.974	
Teacher-student Attention [37]	Normal	0.969	0.989	0.979	0.971	0.990	0.964
	Pneumonia	0.951	0.982	0.966	0.979	0.992	
	COVID-19	0.957	0.877	0.915	0.987	0.963	
	Weighted Avg.	0.961	0.961	0.960	0.977	0.984	
	Macro Avg.	0.959	0.949	0.953	0.979	0.982	
ResGNet-C [30]	Normal	0.942	0.974	0.958	0.946	0.975	0.939
	Pneumonia	0.951	0.855	0.901	0.982	0.944	
	COVID-19	0.910	0.961	0.934	0.971	0.987	
	Weighted Avg.	0.937	0.937	0.936	0.962	0.957	
	Macro Avg.	0.934	0.930	0.931	0.966	0.952	
xViTCOS-CT (Proposed)	Normal	0.997	0.990	0.993	0.997	0.991	0.981
	Pneumonia	0.971	0.982	0.977	0.988	0.993	
	COVID-19	0.960	0.961	0.961	0.988	0.988	
	Weighted Avg.	0.981	0.981	0.981	0.992	0.991	
	Macro Avg.	0.976	0.978	0.977	0.991	0.991	

**FIGURE 2.**
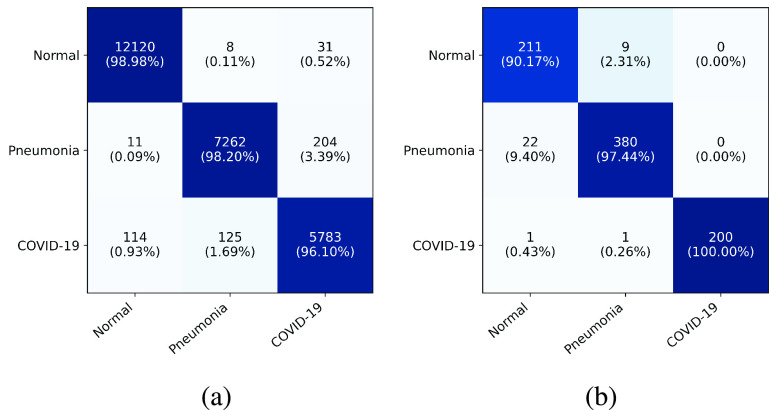
Confusion Matrix: The horizontal and vertical axis consists of the ground true and predicted labels, respectively.

The prowess of the proposed model can be further understood from examining the confusion matrix (Figure 2). The proposed model can distinguish the healthy patients from both covid and non-covid pneumonia cases very efficiently, with an accuracy of almost 99%. Particularly, out of a total of 12245 normal cases, 12120 have been classified correctly, while 11 (0.09%) and 114 (0.93%) cases have been wrongly classified as non-COVID pneumonia and COVID pneumonia classes, respectively. Another interesting point to note here is that while 114 normal cases have been misclassified as COVID-19 and 204 COVID-19 cases have been assigned the non-COVID pneumonia label; the classifier has assigned only 31 COVID-19 originated pneumonia cases a normal class. This implies that the proposed method can distinguish the normal cases from the diseased cases.

#### 2) xViTCOS-CXR

The observations regarding the performance of xViTCOS-CXR compared to its contemporaries are on the same lines as that of xViTCOS-CT, if not better. In terms of classification accuracy, xViTCOS-CXR achieves an accuracy of 96%, outperforming the baseline methods by a considerable margin as can be seen from Table 4. Further, it can be observed that xViTCOS-CXR achieves high recall (100%) and precision values (99%) on the COVID-19 cases, implying that the number of occasions on which the proposed model classified a COVID-19 model as a non-COVID-19 model or vice-versa is extremely low. Examining the entries of Table 4, one can observe that the proposed method is the most balanced in terms of precision-recall when compared with the state-of-the-art baselines. Similarly, we can see that the proposed model attains high specificity and NPV values of almost 100% for the COVID-19 case implying that the number of false positives is almost negligible. This is a valuable characteristic in clinical scenarios since it allows for rapid identification of patients who do not have COVID-19.

**TABLE 4:** Comparison of Performance of xViTCOS-CXR on Chest X-Ray Dataset Against State-of-the-Art Methods

Method	Class Label	Precision	Recall	F1-score	Specificity	NPV	Overall Accuracy
InceptionV3 [85], [86]	Normal	0.932	0.876	0.903	0.974	0.952	0.946
	Pneumonia	0.933	0.964	0.948	0.937	0.967	
	COVID-19	0.990	0.995	0.992	0.997	0.998	
	Weighted Avg.	0.947	0.947	0.946	0.962	0.970	
	Macro Avg.	0.952	0.945	0.948	0.969	0.972	
CoroNet [12]	Normal	0.812	0.923	0.864	0.915	0.967	0.917
	Pneumonia	0.953	0.941	0.947	0.958	0.947	
	COVID-19	1.000	0.865	0.927	1.000	0.958	
	Weighted Avg.	0.924	0.917	0.919	0.956	0.955	
	Macro Avg.	0.922	0.910	0.913	0.958	0.957	
CovidNet [14]	Normal	0.826	0.918	0.870	0.923	0.966	0.919
	Pneumonia	0.950	0.882	0.915	0.958	0.900	
	COVID-19	0.985	0.995	0.990	0.995	0.998	
	Weighted Avg.	0.923	0.920	0.920	0.957	0.943	
	Macro Avg.	0.920	0.932	0.925	0.959	0.955	
Teacher Student Attention [37]	Normal	0.913	0.902	0.908	0.966	0.961	0.932
	Pneumonia	0.918	0.974	0.945	0.922	0.976	
	COVID-19	0.989	0.885	0.934	0.997	0.964	
	Weighted Avg.	0.934	0.932	0.932	0.953	0.969	
	Macro Avg.	0.940	0.920	0.929	0.962	0.967	
MAG-SD [36]	Normal	0.954	0.901	0.927	0.983	0.962	0.951
	Pneumonia	0.931	0.974	0.952	0.935	0.975	
	COVID-19	0.989	0.965	0.977	0.996	0.988	
	Weighted Avg.	0.952	0.951	0.951	0.963	0.974	
	Macro Avg.	0.958	0.947	0.952	0.971	0.975	
xViTCOS-CXR (Proposed)	Normal	0.959	0.902	0.929	0.985	0.962	0.960
	Pneumonia	0.945	0.974	0.959	0.949	0.976	
	COVID-19	0.990	1.000	0.995	0.997	1.000	
	Weighted Avg.	0.959	0.960	0.959	0.971	0.978	
	Macro Avg.	0.965	0.959	0.961	0.977	0.979	

Analysing figure 2b, it can be seen that the class-wise accuracy of COVID-19 is 100%, i.e., all the ground truth COVID-19 cases have been classified as COVID-19, implying that the number of false negatives is zero. This confirms the efficacy of the proposed model in distinguishing between COVID and non-COVID cases.

### D. Qualitative Results

#### 1) Visualization of Feature Space

**FIGURE 3.**
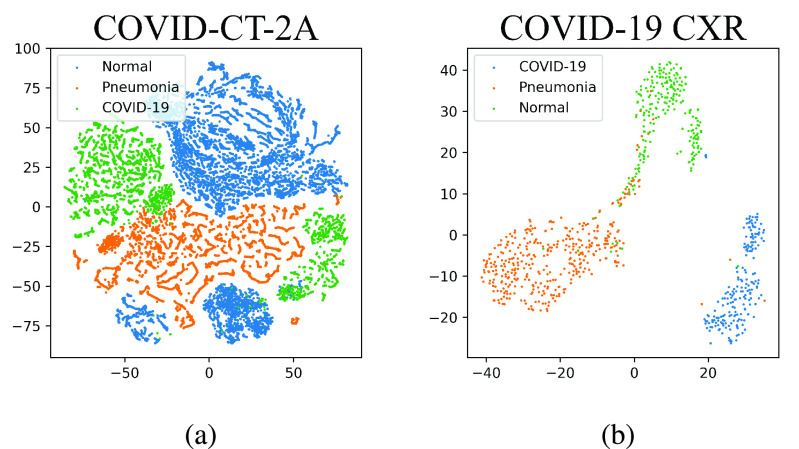
t-SNE plots of penultimate layers of xViTCOS.

To visually analyze how clustered the feature space is, we perform a t-SNE visualization of the penultimate layer’s features for both the models using the test splits. As can be seen from Figure 3, the features in the penultimate layer clusters distinctively for the three different classes.

#### 2) Explainability

**FIGURE 4.**
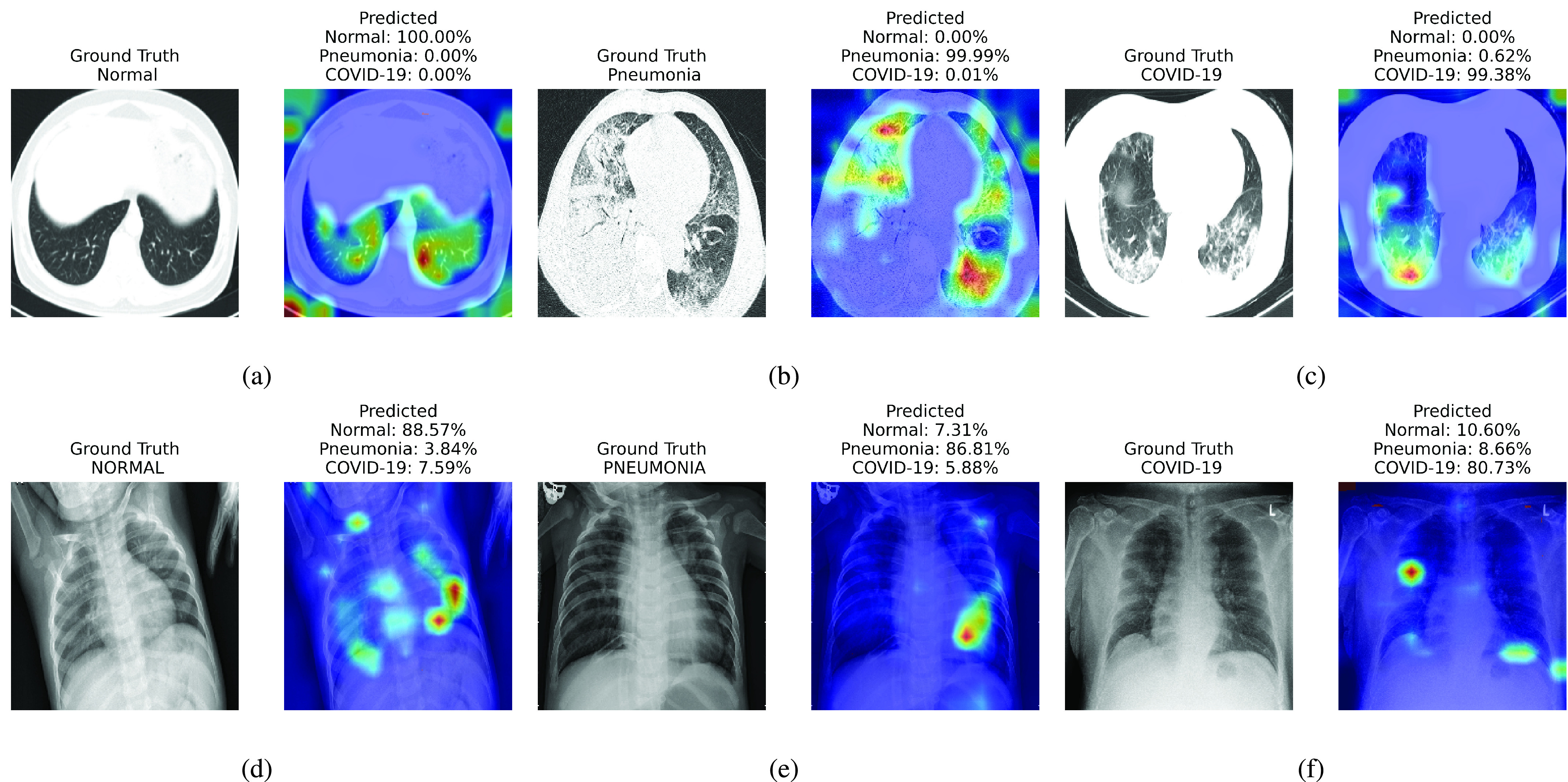
Visualization of different cases (normal, Pneumonia, COVID-19) considered in this study and their associated critical factors in decision making by xViTCOS as identified using the explanability method laid out in [87] for transformers [16]. In each subfigure, the left figure presents the input to xViTCOS and its ground truth label; the right figure presents the predicted probabilities for each class and highlight the factors critical corresponding to the top predicted class. Figure 4a, 4b and 4c corresponds to CT scan and Figure 4d, 4e and 4f corresponds to CXR images.

For qualitative evaluation of xViTCOS we present samples of CXR images and CT scans along with their ground truth labels and corresponding saliency maps along with the prediction in Figure 4. In order to analyse the explainability properties of our proposed method, we use the Gradient Attention Rollout algorithm as outlined in [87]. Further details can be found in Section I of the supplementary document. Figure 4a, 4b and 4c presents CT scans of normal, Pneumonia and COVID-19 cases respectively; Figure 4d, 4e and 4f presents CXR images of normal, Pneumonia and COVID-19 cases respectively.

**FIGURE 5.**
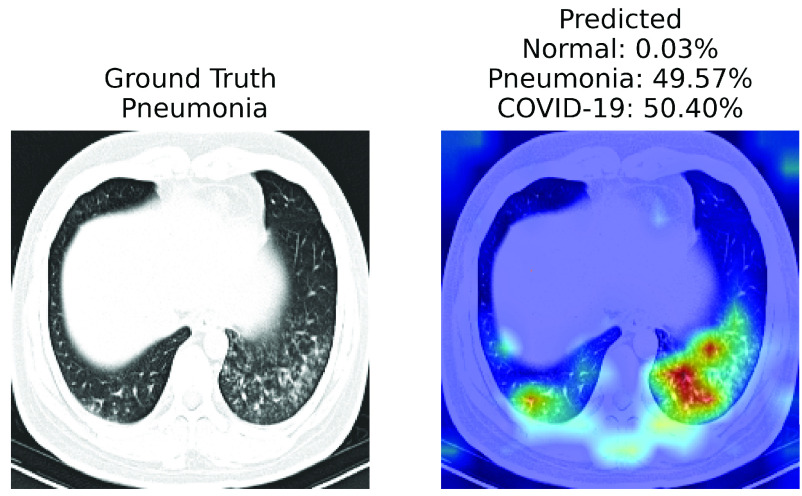
A case of failure. xViTCOS-CT fails to predict the ground truth non-COVID-19 Pneumonia with confidence as it predicts non-COVID-19 Pneumonia with $\approx 50\%$ probability and COVID-19 with $\approx 50\%$ probability. This might happen as the findings on chest imaging in COVID-19 are not exclusive and overlap with many other type of infections [88]. In such cases, human expert intervention is necessary. For a detailed discussion refer to Section V.

Report corresponding to Figure 4b as interpreted by a practicing radiologist: ground glass opacities, consolidation and secondary interlobar septal thickening, in bilateral lung, more extensive in right. xViTCOS-CT correctly highlighted these suspected regions. In Figure 4c xViTCOS-CT localized suspicious lesion regions exhibiting ground glass opacities, consolidation, reticulations in bilateral postero basal lung with subpleural predominance. In Figure 4e Patchy air space opacities noted in right upper and midzone matches the regions highlighted by xViTCOS-CXR. In Figure 4f, radiologist’s interpretation is: thick walled cavity in right middle zone with surrounding consolidation. xViTCOS-CXR is able to correctly identify it. For the cases, where no abnormality is detected (Figure 4a and 4d), xViTCOS focuses on the entire lungs and chest respectively to make a final decision.

## V. Conclusion

In this study, we introduce a novel vision transformer based method, xViTCOS for COVID-19 screening using chest radiography. We have empirically demonstrated the efficacy of the proposed method over CNN based SOTA methods as measured by various metrics such as precision, recall, F1 score. Additionally, we examine the predictive performance of xViTCOS utilizing explanability-driven heatmap plot to highlight the important factors for the predictive decision it makes. These interpretable visual cues are not only a step towards explainable AI, also might aid practicing radiologists in diagnosis. We also analyzed the failure cases of our method. Thus, to enhance the effectiveness of diagnosis we suggest that xViTCOS be used to complement RT-PCR testing. In the next phase of this project, we aim to extend this work to automate the analysis of the severity of infection using vision transformers.

## Supplementary Materials

Supplementary materials
