# Supplementary material for: xViTCOS: Explainable Vision Transformer Based COVID-19 Screening Using Radiography
Source: IEEE J Transl Eng Health Med. 2021 Dec 8;10:1100110. doi: 10.1109/JTEHM.2021.3134096 (PMC8691725; doi:10.1109/JTEHM.2021.3134096)
Supplement: Supplementary materials [file supp1-3134096.pdf]

xViTCOS: Explainable Vision Transformer Based COVID-19 Screening Using Radiography

Supplementary Material

Arnab Kumar Mondal*, Arnab Bhattacharjee*, Parag Singla, and AP Prathosh

I. Explainability

The concept of explainability is subjective and has been a topic of significant discussion in the Machine Learning community. Understanding explainability, in the context of a Vision Transformer used for image classification, boils down to being able to identify the part(s) of an image responsible for classifying it into its corresponding class and what differentiates it from images in other classes. In [1] the authors propose a method to identify class wise attention maps. These maps are able to differentiate between the parts of the image which indicate their affiliation to each individual class. The method used for attaining class wise explainability is called Gradient Attention Rollout. Unlike vanilla Attention Rollout [2], where the attention matrices corresponding to each head is averaged to obtain the final attention map, the authors in [1] propose that while fusing attention heads in every layer, the relevance information from the attention matrices be weighted by the target class attention gradient and then averaged to generate the final map.

In formal terms, let B be the number of blocks in a Transformer model, where each layer is composed of a combination of self attention layers, batch normalization layers, fully connected layers and skip connections. The input to the model is a sequence of s tokens each of dimension d, with a separate token for the classification output, previously defined by [class]. The output is a classification probability vector y of length equal to the number of classes, say C, obtained from the classification head. Each of the h self attention heads focuses on a subspace of length d_h of the total input such that $d_h \times h = d$. The self attention is defined as:

$$\boldsymbol{Att}\left(\boldsymbol{b}\right) = \text{Softmax}\left(\boldsymbol{Query}\left(\boldsymbol{b}\right) \bullet \boldsymbol{Key}\left(\boldsymbol{b}\right)^{T}\right) \quad (1)$$

$$\boldsymbol{Op}\left(\boldsymbol{b}\right) = \boldsymbol{Att}\left(\boldsymbol{b}\right) \bullet \boldsymbol{Val}\left(\boldsymbol{b}\right) \quad (2)$$

where $(\bullet)$ denotes matrix multiplication and $\boldsymbol{Op}\left(\boldsymbol{b}\right) \in \mathbb{R}^{h \times s \times d_h}$ is the output of the attention module in block b. $\boldsymbol{Att}\left(\boldsymbol{b}\right) \in \mathbb{R}^{h \times s \times s}$ is the attention map of block b where each row represents the attention coefficient of each token with respect to the row's token. Let $\nabla \boldsymbol{Att}\left(\boldsymbol{b}\right)$ be the gradients of $\boldsymbol{Att}\left(\boldsymbol{b}\right)$ with respect to a target class t and $\boldsymbol{R}\left(\boldsymbol{nb}\right)$ be the relevance, where nb represents that the softmax layer is the $n-th$ layer of $b-th$ block. Then the final output $\boldsymbol{A_F} \in \mathbb{R}^{s \times s}$ is defined as follows:

$$\hat{\boldsymbol{A}}\left(\boldsymbol{b}\right) = \boldsymbol{I} + \mathbb{E}_h\left[\nabla \boldsymbol{Att}\left(\boldsymbol{b}\right) \odot \boldsymbol{R}\left(\boldsymbol{nb}\right)\right] \quad (3)$$

$$\boldsymbol{A_F} = \hat{\boldsymbol{A}}\left(\boldsymbol{1}\right) \bullet \hat{\boldsymbol{A}}\left(\boldsymbol{2}\right) \cdots \hat{\boldsymbol{A}}\left(\boldsymbol{B}\right) \quad (4)$$

where $\odot$ is the Hadamard Product and $\mathbb{E}_h$ is the expectation across the dimension of attention heads. Only the positive values of the gradient- relevance multiplication are kept and all negative values are brought up to zero. The identity is added to account for the skip connections in the Transformer block.

II. Experimental Results

A. ViT Architecture Search

In this section, we present the results of using different ViT architectures for classification on the Chest X-Ray Dataset. The architectures used in this analysis are those proposed in [3] and [4]. In both these references the authors propose two basic kinds of Transformer architectures, namely the BASE and the LARGE architectures. The BASE architecture, first proposed in [4], had a similar structure to that of OpenAI's Generative Pre-trained Transformer(GPT) [5]. The authors had done so to make fair comparison between $BERT_{BASE}$ and OpenAI's GPT models on a multiple language modeling tasks. Let H be the number of hidden layers, A be the number of attention heads and L be the number of encoder layers in a transformer. The BASE architecture corresponds to the following configuration: $(L = 12, H = 768, A = 12)$. The LARGE architecture corresponds to the following configuration:$(L = 24, H = 1024, A = 16)$. Also, depending on the size of the image patches that are given as input to the transformer, these architectures are further classified into subgroups. A Transformer model having a BASE architecture and taking image patches of shape 16×16 as inputs is denoted by ViT-B/16. Similarly three other architectures are defined namely, ViT-B/32, ViT-L/16 and ViT-L/32. We follow the same naming convention as in [3]. The classification performance of these four architectures are given in Table I.

All the authors are with Indian Institute of Technology Delhi, New Delhi 110016, India. Email: anz188380@iitd.ac.in, arnab.bhattacharjee@uqidar.iitd.ac.in, parags@iitd.ac.in, prathoshap@iitd.ac.in.

* indicates equal contribution.

TABLE I: ViT Architecture Search

Method	Class Label	Precision	Recall	F1-score	Specificity	NPV	Overall Accuracy
xViTCOS-CXR (B/16)	Normal	**0.959**	0.902	0.929	0.985	0.962	0.960
	Pneumonia	0.945	0.974	0.959	0.949	0.976	
	COVID-19	0.990	**1.000**	0.995	0.997	**1.000**	
xViTCOS-CXR (B/32)	Normal	0.945	0.88	0.912	0.979	0.954	0.951
	Pneumonia	0.940	0.969	0.955	0.945	0.972	
	COVID-19	0.980	**1.000**	0.990	0.994	**1.000**	
xViTCOS-CXR (L/16)	Normal	0.956	**0.920**	**0.937**	0.983	**0.968**	**0.965**
	Pneumonia	**0.955**	0.974	**0.964**	**0.959**	0.976	
	COVID-19	**0.995**	**1.000**	**0.998**	**0.998**	**1.000**	
xViTCOS-CXR (L/32)	Normal	0.948	0.799	0.880	**0.993**	0.926	0.937
	Pneumonia	0.913	**0.990**	0.950	0.915	**0.990**	
	COVID-19	0.979	0.995	0.971	0.982	0.998	

TABLE II: Ablation Studies for xViTCOS-CXR: Impact of multi-stage transfer

Method	Class Label	Precision	Recall	F1-score	Specificity	NPV	Overall Accuracy
Training ViT from scratch on COVID-19 CXR data	Normal	0.754	0.444	0.559	0.942	0.811	0.710
	Pneumonia	0.688	0.897	0.779	0.634	0.873	
	COVID-19	0.740	0.655	0.694	0.926	0.893	
Training ViT from scratch on CheXpert and finetuning on COVID-19 CXR data	Normal	0.777	0.641	0.702	0.927	0.866	0.821
	Pneumonia	0.819	0.882	0.849	0.824	0.886	
	COVID-19	0.867	0.915	0.891	0.955	0.972	
No intermediate finetuning on CheXpert	Normal	0.894	0.906	0.900	0.957	**0.962**	0.943
	Pneumonia	0.944	0.946	0.945	**0.949**	0.952	
	COVID-19	**1.000**	0.980	0.990	**1.000**	0.994	
xViTCOS-CXR (multi-stage transfer)	Normal	**0.959**	0.902	**0.929**	**0.985**	**0.962**	**0.960**
	Pneumonia	**0.945**	**0.974**	**0.959**	**0.949**	**0.976**	
	COVID-19	0.990	**1.000**	**0.995**	0.997	**1.000**	

TABLE III: Ablation Studies for xViTCOS-CXR: Impact of freezing layers.

Method	Class Label	Precision	Recall	F1-score	Specificity	NPV	Overall Accuracy
Only the final classification head was trained	Normal	0.904	0.846	0.874	0.964	0.940	0.921
	Pneumonia	0.913	0.936	0.924	0.919	0.941	
	COVID-19	0.956	0.980	0.968	0.985	0.993	
First nine encoders of ViT were frozen	Normal	0.908	0.885	0.896	0.964	0.954	0.938
	Pneumonia	**0.949**	0.951	0.950	0.954	0.956	
	COVID-19	0.951	0.975	0.963	0.984	0.992	
First six encoders of ViT were frozen	Normal	**0.961**	0.846	0.900	**0.986**	0.942	0.945
	Pneumonia	0.920	**0.979**	0.949	0.924	**0.980**	
	COVID-19	0.980	0.995	0.987	0.993	0.998	
First three encoders of ViT were frozen	Normal	0.919	**0.927**	0.923	0.968	**0.971**	0.953
	Pneumonia	0.958	0.954	0.956	**0.963**	0.959	
	COVID-19	0.980	0.980	0.980	0.993	0.993	
xViTCOS-CXR (All the layers were finetuned)	Normal	0.959	0.902	**0.929**	0.985	0.962	**0.960**
	Pneumonia	0.945	0.974	**0.959**	0.949	0.976	
	COVID-19	**0.990**	**1.000**	**0.995**	**0.997**	**1.000**	

TABLE IV: Ablation Studies for xViTCOS-CT: Impact of freezing layers.

Method	Class Label	Precision	Recall	F1-score	Specificity	NPV	Overall Accuracy
First three encoders frozen	Normal	0.99	**0.994**	0.992	0.991	**0.995**	0.972
	Pneumonia	**0.987**	0.933	0.959	**0.995**	0.974	
	COVID-19	0.922	0.975	0.948	0.974	**0.992**	
First six encoders frozen	Normal	0.995	0.989	0.992	0.996	0.990	0.978
	Pneumonia	0.958	**0.988**	0.973	0.983	**0.995**	
	COVID-19	**0.97**	0.945	0.957	**0.991**	0.983	
First nine encoders frozen	Normal	0.991	0.983	0.987	0.992	0.984	0.972
	Pneumonia	0.966	0.972	0.969	0.986	0.989	
	COVID-19	0.939	0.949	0.944	0.981	0.984	
xViTCOS-CT	Normal	**0.997**	0.990	**0.993**	**0.997**	0.991	**0.981**
	Pneumonia	0.971	0.982	**0.977**	0.988	0.993	
	COVID-19	0.960	**0.961**	**0.961**	0.988	0.988	

The transformer model performs the best with the L/16 architecture with an accuracy of 96.5%, followed by the B/16 architecture which attains an accuracy of 96%. However, the increase in the computational expense in moving from B/16 to L/16 is exceptionally high. Hence a trade off was made and the B/16 architecture was used for all the future experimentation in this paper. Also, the B(L)/32 architectures didn't fare well as compared to their 16×16 input sized counterparts. The patch size is an important hyperparameter that decides not just how much information from an image goes into making a token

which constitute the input sequence but also decides the input sequence length. If the patch size is too small, the number of constituents of the input sequence can grow quadratically. If the size is too large, the patch can constitute of diverse, coarser and possibly incomplete information instead of just relevant local information, implying that the representative tokens that would be generated from them would not be good representations of the local features of the actual image. This is possibly the case in our example as well.

B. Ablation Studies on CXR Dataset

To understand the contributions made by each of the proposed training (finetuning) steps in training xViTCOS-CXR, we conduct several ablation experiments in this section. Table II presents the results. When ViT is trained on COVID-19 CXR data, its performance is worst as the dataset has very less training samples. CheXpert [6] consists of 224,316 chest radiographs of 65,240 patients. However, these many images are not sufficient for training ViT from scratch. Therefore, although the performance of the model improves, it is not comparable to the SOTA results. When the ViT model pre-trained on imagenet is directly used for finetuning on CXR dataset, we see a huge boost in the performance. However, the best performance is achieved when the training procedure involves an intermediate finetuning step using CheXpert [6]. We may conclude that the intermediate finetuning helps the model learn useful features related to chest X-ray.

In order to analyse the effects of freezing a subset of layers on the classification performance of the proposed model, we conduct three experiments by subsequently freezing the first three, six and nine encoder layers of the model finetuned on the CheXpert data. These models are then trained on the COVID-19 CXR dataset. A fourth experiment is conducted where only the classification head of the model is allowed to train on the Covid CXR images and all the remaining layers are frozen, following the intermediate finetuning on CheXpert. The results are shown in Table III. As expected freezing more layers during training on the CXR dataset leads to decreasing accuracy of classification, with the model where none of the layers were frozen performing the best amongst the lot and the model where only the classification head is trained performs the worst. This implies that the more the number of trainable layers, the more is the capacity leading to a better performance of xViTCOS-CXR.

Similar ablation studies have been conducted on xViTCOS-CT and the results and detailed analysis has been added in the supplementary. Since for training xViTCOS-CT, only a single stage of transfer learning was sufficient, we conducted only the incremental encoder layer freezing experiments to understand the contribution of multiple stacked encoder layers.

C. Ablation Studies on CT-Scan Dataset

For xViTCOS-CT, only a single stage of transfer of weights was required during training owing to the presence of a sufficient number of datapoints from the Covid-CT 2A dataset to train the transformer. Hence in the ablation studies only the impact of freezing the encoder layers has been studied. As can be seen from Table IV, the proposed algorithm performs the best when all of its encoder layers are available for training. Although, even with a few of the trainable encoders, the degradation in performance is not too significant.

References

[1] H. Chefer, S. Gur, and L. Wolf, "Transformer interpretability beyond attention visualization," in *Proc. of CVPR*, 2021.

[2] S. Abnar and W. Zuidema, "Quantifying attention flow in transformers," 2020.

[3] A. Dosovitskiy, L. Beyer, A. Kolesnikov, D. Weissenborn, X. Zhai, T. Unterthiner, M. Dehghani, M. Minderer, G. Heigold, S. Gelly, J. Uszkoreit, and N. Houlsby, "An image is worth 16x16 words: Transformers for image recognition at scale," in *Proc. of ICLR*, 2021.

[4] J. Devlin, M.-W. Chang, K. Lee, and K. Toutanova, "BERT: Pre-training of deep bidirectional transformers for language understanding," in *Proceedings of the 2019 Conference of the North American Chapter of the Association for Computational Linguistics: Human Language Technologies, Volume 1 (Long and Short Papers)*, pp. 4171–4186, June 2019.

[5] A. Radford, K. Narasimhan, T. Salimans, and I. Sutskever, "Improving language understanding by generative pre-training," *OpenAI Blog*, 2018.

[6] J. Irvin, P. Rajpurkar, M. Ko, Y. Yu, S. Ciurea-Ilcus, C. Chute, H. Marklund, B. Haghgoo, R. L. Ball, K. Shpanskaya, J. Seekins, D. A. Mong, S. S. Halabi, J. K. Sandberg, R. Jones, D. B. Larson, C. P. Langlotz, B. N. Patel, M. P. Lungren, and A. Y. Ng, "Chexpert: A large chest radiograph dataset with uncertainty labels and expert comparison," in *Proc. of AAAI*, 2019.
